# STRESS, STEM CELLS, AND THE GUT MICROBIOME—IMPLICATIONS FOR AGING

**DOI:** 10.1093/geroni/igad104.0384

**Published:** 2023-12-21

**Authors:** Imilce Rodriguez-Fernandez

**Affiliations:** University of Puerto Rico Rio Piedras, San Juan, Puerto Rico, United States

## Abstract

Adult somatic stem cells have a crucial role in maintaining the regenerative capacity of tissues throughout the lifespan of the organism. With age comes a reduction in tissue renewal and repair upon injury. Understanding the mechanisms that maintain stem cell function could potentially lead to the development of therapeutic strategies to mitigate age-related tissue degeneration and improve healthspan. Of particular interest is understanding how stem cells deal with different types of stressors such as oxidative stress and bacterial stress. To study this, we use the Drosophila intestinal stem cells (ISCs) as a model system. ISCs are normally quiescent but during stress, they divide asymmetrically to give rise to one stem cell and one differentiated cell: an enteroblast (EB) that will become an Enterocyte (EC) or an enteroendocrine progenitor (EEp) cell that will later become a mature enteroendocrine (EE) cell. ECs have roles in digestion and immune response. EEs have roles in hormones and peptide secretion. ISCs have a crucial role in regenerating lost intestinal cells after bacterial infection, oxidative stress, or normal cell attrition. Thus, ISCs are always sensing the gut environment and respond to cues by activating or repressing proliferation. By studying the function of two important stress-sensing transcriptions namely Nrf2/CncC and HSF1 in ISCs, we have uncovered an unprecedented role of these genes in stem cell identity and the regulation of the gut microbiome. These findings could shed light on the mechanisms altered during aging that contribute to stem cell mis-differentiation and microbial dysbiosis.

